# Introduction of Nurse-Led Rehabilitation Services for Patients With Stroke After Discharge to Improve Self-Care Management in Bangladesh: Pilot Randomized Controlled Trial

**DOI:** 10.2196/88808

**Published:** 2026-07-17

**Authors:** Salma Akhter, KATM Ehsanul Huq, Shota Date, Mahmudul Islam, Farida Khatun Chhobi, SK Jakaria Been Sayeed, Md Mazharul Islam, Mahabuba Afrin, Kana Kazawa, Michiko Moriyama

**Affiliations:** 1 Graduate School of Biomedical and Health Science Hiroshima University Hiroshima, Hiroshima Japan; 2 National Institute of Neurosciences & Hospital Dhaka, Dhaka Division Bangladesh

**Keywords:** caregiver burden, post discharge, rehabilitation, self-care management, stroke

## Abstract

**Background:**

Stroke is a leading cause of disability and death, resulting in a clinical, social, and economic burden upon families and the health system worldwide.

**Objective:**

This study aimed to introduce nurse-led rehabilitation services for patients after stroke to improve functional independence for self-care management.

**Methods:**

A pilot, open-label, 2-arm (1:1), randomized controlled trial was conducted at the National Institute of Neuroscience & Hospital in Bangladesh between March and August 2025. A total of 64 patients with stroke were enrolled using simple randomization. In the intervention group (IG), patients received rehabilitation education and assistive devices to improve activities of daily living. The control group (CG) received usual hospital care for stroke. Data were collected through a structured questionnaire. After discharge from the hospital, study nurses enrolled patients after stroke after a face-to-face assessment and provided rehabilitation education at the hospital and monthly rehabilitative education at home for 3 months. The study outcomes were improvement of functional independence, self-efficacy, social participation and reduction of caregivers’ burden.

**Results:**

Among 64 participants, 47 (73%) completed the study. Results depict an increase in self-care capacity, self-efficacy, social involvement and a decrease in care burden in both groups from baseline to end line; nevertheless, there was no statistically significant difference between IG and CG (*F*_2, 44_=0.113; *P*=.74). Functional Independence Measure mean scores (IG: mean 78.4, SD 37.6; CG: mean 74.3, SD 35.4; *P*=.70; 95% CI –25.16 to 18.14), self-efficacy (IG: mean 27.0, SD 6.9; CG: mean 26.6, SD 7.1; *P*=.83; 95% CI –3.6 to 4.6), social participation (IG: mean 30.4, SD 21.4; CG: mean 28.2, SD 17.2; *P*=.61; 95% CI –9.33 to 13.61), and care burden (IG: mean 24.9, SD 13.6; CG: mean 25.2, SD 12.6; *P*=.83; 95% CI –8 to 7). In qualitative analysis, we noticed enhanced self-care capacities of patients with poststroke disabilities after rehabilitative intervention, as indicated by participants’ perceptions.

**Conclusions:**

In this study, patients’ self-care capability was improved after the intervention from baseline, and improvements were noted by using assistive devices based on patients’ perceptions and responses. The study findings demonstrated the importance of early self-care management for patients with poststroke disabilities. Future research should focus on long-term, community-integrated strategies that involve primary care, enhanced technological support, and caregiver-focused disability adjustment programs to achieve better outcomes.

**Trial Registration:**

ClinicalTrials.gov NCT06786559; https://clinicaltrials.gov/search?id=NCT06786559

## Introduction

Stroke is a leading cause of disability and death, particularly in low- and middle-income countries (LMIC) [1]. Notably, about 86% of stroke-related deaths and 89% of stroke-associated disabilities have occurred in LMIC. The causes are mainly a lack of prevention, acute management, and rehabilitation facilities and services [2]. The prevalence of stroke globally was 1.2%; in developed countries, 3%; and in developing countries, in 2021 [3]. In Bangladesh, stroke prevalence was 1.1% in 2018 [4], and the trend is rising due to an increase in hypertension, diabetes, smoking, and other modifiable risk factors. High mortality and disability are driven by poor access to acute interventions and limited rehabilitation [5]. Unfortunately, developing countries lack a national database system; therefore, the occurrence of stroke is often underreported [6].

Stroke rehabilitation is a multidimensional process provided by multidisciplinary teams, including physicians, physical and occupational therapists, nurses, psychologists, and social workers [6-8]. It encompasses physical rehabilitation [6], occupational therapy [7], and speech and language therapy [9]. Rehabilitation also includes cognitive therapy for improvement of memory and executive deficits [10], psychological care for poststroke depression and anxiety [11], and social/vocational programs for reintegration of patients into society [6].

The *International Classification of Functioning, Disability, and Health* (*ICF*) is the World Health Organization (WHO) framework for measuring functioning disabilities in the environment where patients with disabilities act and are involved in social participation [12]. *ICF* emphasizes the importance of environmental factors, including assistive devices, which enable persons with impaired function to conduct daily activities and participate in society [12]. However, assistive devices are often not available and expensive, so patients cannot afford specially in LMIC [13]. Acute and postacute rehabilitation services are crucial for improving the quality of life for stroke survivors and their caregivers [14]. To mitigate stroke-related disability and death, urgent priorities include scaling up prevention strategies, improving acute care capacity, and facilitating community-based rehabilitation to address the growing burden of stroke in situations where acute and postacute rehabilitation services are not accessible in health care facilities [1].

In LMIC, including Bangladesh, there is a lack of institutional acute, postacute, and long-term rehabilitation services available after receiving acute care management from the hospital [15]. Patients with disabilities who need rehabilitation face inadequate health care infrastructure and financial resources [16]. Moreover, poststroke function analysis is a lengthy process that begins with impairment evaluation, rehabilitation training, recovery monitoring, and providing the necessary support to carry out daily tasks [17]. Bangladesh faces a critical shortage of rehabilitation workforce compared to the number of patients with disabilities [18]. Rehabilitation services are limited and fragmented, concentrated in tertiary hospitals, where nongovernment organizations, such as the Centre for the Rehabilitation of the Paralyzed (CRP) and Bangladesh Rural Advancement Committee, provide community-based rehabilitation in some parts of the country [19]. As a result, patients with disabilities have no choice but to be cared for by nonprofessional family members in a nonsupportive and unequipped living environment, which makes it difficult to support their independence and participation in society [20]. Moreover, caregivers face significant challenges, including limited stroke knowledge, financial strain, psychological stress, and cultural expectations [21].

As there are insufficient poststroke institutional services available in Bangladesh, home-based stroke rehabilitation could be an alternative way to play a vital role in ensuring continuity of care and supporting functional recovery within patients’ home environments [22]. Globally, it is delivered through multidisciplinary outreach teams, primary care providers, caregiver involvement, and increasingly telerehabilitation, which has proven effective in functional and activities of daily living (ADL) recovery [6,7,23].

**Figure 1:**
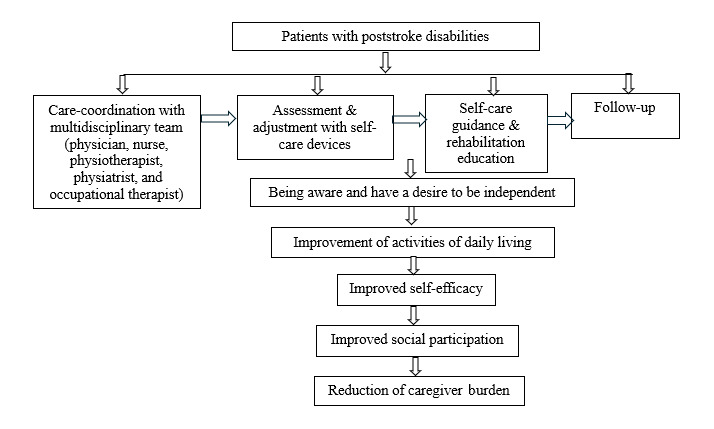
Study conceptual framework.

Nurse-led rehabilitation is well-practiced globally [24]. They excel at coordinating necessary services and providing rehabilitation care integrated with disease management education for patients and their families. However, in Bangladesh, the role of nurses is limited to acute care nursing, and they do not participate in providing rehabilitation services to patients with poststroke disabilities. Therefore, the purpose of this study was to test the feasibility and efficacy of nurse-led rehabilitation programs focused on improving ADL among patients after stroke in Bangladesh (Figure 1). 

## Methods

### Study Design and Participants

This was a parallel (1:1), open-label, prospective, randomized controlled trial that adhered to the CONSORT (Consolidated Standards of Reporting Trials) guidelines (Multimedia Appendix 1) [25]. In addition to evaluating the intervention quantitatively, we qualitatively explored the feasibility of home-based rehabilitation and the use of assistive devices for promoting functional independence among patients after stroke and their family caregivers. This study was conducted at the National Institute of Neurosciences & Hospital (NINS&H) in Dhaka, Bangladesh, between March 2025 and August 2025. As NINS&H is the only specialized referral hospital in Bangladesh, most of the patients with stroke are usually discharged within 15 days to 1 month due to overcrowding/high bed occupancy. Therefore, we included patients who were admitted within 5-14 days of the onset of their stroke and discharged within 1 month from the hospital. Patients with stroke and their family caregivers, both male and female individuals, aged ≥18 years, were included. Patients were enrolled irrespective of their time (first or any recurrent) and type (ischemic or hemorrhagic) of stroke, with a modified Rankin scale (mRS) score of 1-4. Patients who had a physician’s advice for rehabilitation therapy and needed assistive devices for ADL were included. It is evident that within this time (1-2 weeks), if rehabilitation is initiated, the patient experiences a better prognosis [26]. We conveniently enrolled only participants living in the Dhaka district to ensure follow-up visits with minimal dropout.

### Sample Size

Sample size was generated using statistical power of 80% at α=.05 (2-tailed), with an estimated medium effect size of 0.75 [27]. We used this effect size as we had sufficient power to detect a meaningful effect. The sample size was calculated to be 29 for each arm (a total of 58 for both arms). Considering a 10% dropout rate, the estimated total sample size was 64 (32 in each arm). We used G*Power software (version 3.1.9.4; Heinrich Heine University Düsseldorf) for Windows for this calculation.

### Randomization and Allocation

Participants were randomized equally into intervention and control groups (CGs; n=32 in each group) using a simple random technique by computer-generated numbers, regardless of their age and sex. Participants were sequentially allocated to the intervention and CGs based on the randomization chart. Randomization was done, and the random allocation sequences were kept by a researcher who was not involved in this study to minimize bias.

### Assessment of Eligibility and Recruitment Procedures

At first, patients who were admitted to the hospital were assessed by physicians and the research team to identify any physical impairment. The physiotherapist also assessed the range of motion and notified the physician who required rehabilitation based on their disabilities. After checking the eligibility criteria and verifying with the physician, research assistant (RA) nurses explained the study purpose, ensured home visits for follow-up, and obtained written informed consent from patients and their caregivers. The patient was assessed for functional independence using the Functional Independence Measure (FIM) by RA nurses to evaluate their level of functioning. They also assessed the patient’s self-efficacy, social participation, and caregivers’ burden.

### Interventions

#### Nurse-Led Intervention

Intervention nurses assessed patients’ needs, prepared and supported the use of assistive devices, provided rehabilitation education to patients and carers, used telenursing to monitor patients’ recovery remotely, and coordinated multidisciplinary care. This comprehensive nurse-led approach empowered the stroke survivors to participate actively in their recovery, promoted independence in daily activities, reduced complications and carer burden, and ultimately improved self-care management.

#### Rehabilitation Education

Rehabilitation education was provided according to the researcher-developed health booklet, followed by established guidelines and textbook [28] and a published journal guideline [29]. This booklet includes environmental adjustment, self-care management guidelines with disability (eg, eating and mouth care, grooming, bathing, toileting, and mobility), tips to avoid falling, instructions to use assistive devices, changing dietary habits, health monitoring, strategies to manage stress, exercise instructions, and a daily exercise and medication intake record chart (Multimedia Appendix 2).

#### Assistive Devices

The research team provided assistive devices to improve ADL (eg, spoon holder, bottle opener, pen holder, and cup/mug holder) using a 3D printer and physical improvement–aiding instruments (eg, special adjustable spoon, walking cane, exercise ball, Thera belt, and massage ball) to help patients with poststroke disabilities with self-care. The 3D printers were used to produce the assistive devices, and these were designed and developed by an occupational therapist and adjusted according to the needs of patients with disabilities.

#### For the Intervention Group

After assessment, patients and caregivers were trained (for 30 minutes during their discharge from the hospital) in self-care with stroke disabilities (eg, eating, grooming, bathing, dressing, and toileting), physical exercise, lifestyle modification [28,29], and prevention of complications after stroke through a researcher-developed “Health booklet” by the RA nurses. The booklet was provided to patients to follow at home for daily practice and a record of exercise with medication intake. Based on their disabilities, adaptive and exercise devices were provided for use at home to promote functional independence. After discharge from the hospital, they were followed by RA nurses through home visits and teleservices biweekly for 3 months (a total of 5 times). The telehealth services include conversations with patients and caregivers and follow-up on adherence to the use of assistive devices provided by RA nurses. These services follow rehabilitation guidelines and health booklet-guided exercises for improving muscle strength. If needed, guidance on the use of assistive devices was also provided through video calls. During these follow-up visits, they assessed patients’ regular self-care activities, adjustment to devices, exercise and adaptive behaviors. During the final follow-up, after 3 months of the initial assessment, researchers and RAs evaluated patient outcomes at the hospital. If the patient was unable to attend the hospital, a home visit was made for follow-up.

#### For the CG

The patients received the usual care from the RA nurses and physician to which they were entitled. They received hospital advice on treatment adherence for stroke prevention during discharge. During the 3 months, patients were requested to visit the hospital for follow-up visits and data collection. We were provided with a health booklet for all and assistive devices who needed after the final evaluation for ethical purposes.

### Preparation for Data Collection

#### Making a Multidisciplinary Team

Upon starting the study, researchers formed a multidisciplinary team consisting of a physician, RA nurses, a physiotherapist, and an occupational therapist under the supervision of the co–principal investigator (neurologist). They collaborated to assess patients from recruitment through final follow-up for evaluation.

#### Training the Study Nurses

A total of 7 RA nurses, who were involved in this study, received 1 week of training through lectures and practice on self-care, physical exercise, mobility, and telenursing services by the principal investigator based on the health booklet. They also received protocol-specific hands-on training for data collection, performing study procedures about self-care, behavioral changes, including adaptation and motivation, and assembling assistive devices for patients with disabilities using a 3D printer. They had regular refresher sessions about the enlisted activities with the principal investigator before and during the study period.

### Data Collection Procedures

After allocating study participants to either the intervention group (IG) or the CG, the RA nurses conducted a face-to-face interview to collect data about the sociodemographic characteristics and health conditions of stroke survivors and their family caregivers. At baseline (T0), the functional independence measurement, self-efficacy, social participation, and care burden of the family were assessed. The RA nurses periodically followed up with the patients of the IG through home visits (twice a month) and teleservice (twice a month) to check their motivation and cultural values to adjust to the intervention devices and health booklet.

In the community, the nurse visited the home setting to observe the home environment and adjust to patients’ needs. If required, they suggested modifications to home settings. Moreover, they assessed the way of implementing assistive devices for performing ADL and rehabilitation for improving muscle strength. Nurses demonstrated the use of assistive devices if needed. They also demonstrated the exercise procedure using the health booklet and motivated patients and family caregivers to maintain the daily record book.

To prevent participant contamination, RA nurses obtained consent and collected data in 2 different rooms. They also maintained different schedules for the IG and CG. As the same RA nurses collected data from both groups, they strictly maintained confidentiality to share the intervention procedures with the CG. For home-based data collection, RA nurses were divided into the IG and CG.

To evaluate the feasibility and chronological changes of adjustment of home-rehabilitation and assistive devices qualitatively, RA nurses interviewed participants through some open-ended questions and observed participants of the IG every time they contacted them. The interview was conducted for 20-30 minutes by RA nurses based on observation points and interview questions during home-based follow-up. The observation and interview points were as follows: “home environment, adjustment, motivation and use of assistive devices, lifestyle changes pattern with risk factors, level of required assistance after using assistive devices, adherence to rehabilitation regimen, mental status, communication, and social participation” after having a stroke (Multimedia Appendix 3).

### Outcome Measures

#### Primary End Point: Functional Independence

Functional independence was assessed using the FIM scale. The FIM was developed in 1983 by a task force from the American Congress of Rehabilitation Medicine and the American Academy of Physical Medicine and Rehabilitation [30]. The Bengali version of the scale was implemented among the patients with functional disabilities in Bangladesh and found to be valid and reliable (α=.97) [31]. This scale has 18 items from level 1 (total assistance) to level 7 (complete independence). This scale consists of 2 subscales, including physical (13 items) and cognitive (5 items) domains. The physical domain score ranges from 13 to 91, and the cognitive domain score ranges from 5 to 35. The range of the total score is from 18 to 126. The higher the score, the better the level of independence.

#### Secondary End Points

##### Self-Efficacy

Self-efficacy was assessed by the Generalized Self-Efficacy Scale [32]. This scale assessed patients’ self-efficacy after experiencing poststroke stress and the strength of an individual’s beliefs in situations. It was translated into the Bengali version and was found valid and reliable (α=.7 to .91) [33]. It has a total of 10 items with a 4-point Likert scale that ranges from 1=not at all true to 4=exactly true. The lowest score is 10, and the highest score is 40. The higher score indicates better self-efficacy.

##### Social Participation

Social participation was measured using the Participation Scale based on the WHO *ICF* domain for assessing a person’s interaction and participation in community life, which measures the severity of social participation restrictions. They standardized the Bengali version of the scale and found it to be valid and reliable (α=.91) [34]. The score ranges from 0 to 70. The higher the score, the worse the level of participation.

##### Caregiver Burden

Care burden was assessed by the Zarit Caregiver Burden Assessment (12-item) scale, short version, to assess the subjective burden of informal caregivers of patients with chronic diseases [35]. The Bengali version of the scale was implemented among patients with stroke in Bangladesh and was found valid and reliable (α=.85) [36]. The score ranges from 0 to 48. No cutoff point was assigned for classifying the care burden; a higher score indicated a higher care burden.

#### Qualitative Observations

We qualitatively analyzed the assistive device-use rate, motivation to use, and unmet needs for physical improvement among participants who received our intervention (IG).

### Analysis

To compare data between the 2 groups at different time points, we conducted a per-protocol set analysis to see the actual efficacy of the intervention. For baseline data, descriptive statistics were used after the normality test. The normal data distribution was examined using Shapiro-Wilk’s test, histograms, box plots, and normal Q-Q plots for both the control and IGs, with *P*>.05. We used chi-square or the Fisher exact test to compare the IG and the CG using categorical variables, which were reported as frequencies and percentages. Since our data were not normally distributed, we performed the Mann-Whitney *U* test to report the continuous variables as the mean (SD). The mean differences between the 2 groups were compared by an independent *t* test at each time point (T0 and T1). In addition, to assess changes in outcomes within the groups over time, a 2-way repeated-measure ANOVA was used. The level of significance was set at *P*<.05. We conducted generalized linear models considering covariates such as age, residence, and mRS to examine the influence of additional rehabilitation activities (home-based and CRP). We used SPSS (version 31.0; IBM Corp). For the qualitative analysis, after transcribing and translating, we converged and coded the statements. Then we used Dedoose software (version 10.0.59; Socio Cultural Research Consultants, LLC), and the results were categorized into themes and subthemes.

### Ethical Considerations

This study was conducted following the ethical principles of the Declaration of Helsinki after getting ethical approval from the institutional review board of NINS&H (approval number IRB/NINS/2024/392), Dhaka, Bangladesh. After an explanation of the study objective, procedure, risk, and benefit, written consent was obtained from participants and their caregivers. Patients’ privacy and confidentiality were maintained throughout the study, and withdrawal was ensured without any consequences. Data were deidentified and assigned unique identification numbers for analysis, and no personal information was used in any publication or presentation resulting from this research. Data were stored and accessible by the principal investigator on a password-protected personal computer.

## Results

### Overview

**Figure 2:**
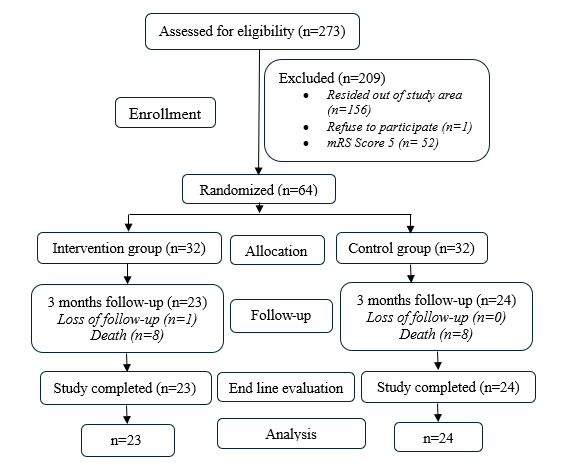
Study flowchart based on the CONSORT (Consolidated Standards of Reporting Trials) statement. mRS: modified Rankin scale.

A total of 273 participants were evaluated to determine their eligibility for the study. Among them, 209 of 273 did not meet the inclusion criteria; 156 of 273 resided out of the study area, 528 of 273 had an mRS score of 5, and 1/273 refused to participate in this study. Therefore, a total of 64 patients were enrolled equally in both groups (IG: n=32 and CG: n=32); 47 (73%) completed (IG: n=23 and CG: n=24) the study, and were analyzed. Among the participants who did not complete the study (n=17), 16/64 (25%) died during the first month of intervention (IG: n=8 and CG: n=8), and 1/64 (1.5%) was lost to follow-up (Figure 2).

### Sociodemographic and Clinical Characteristics of the Participants

The mean age of participants was 55.6 (SD 14.4) years, with a range of 20-82 years. Most of the participants were male (n=52, 81%) and resided in urban areas (n=55, 86%). A higher proportion of rural residents was noted in the CG (n=7, 22% vs n=2, 6%) compared to the IG.

Overall, no significant differences were observed between the IG and CG across sociodemographic and clinical characteristics and for comorbidity, indicating successful randomization (Table 1).

Preintervention FIM scores were compared with sociodemographic and clinical characteristics in Multimedia Appendix 4. The participants’ age, religious status, residence, tobacco smoking, and mRS were significantly associated with FIM (all *P*<.05).

**Table 1:** Sociodemographic and clinical characteristics of the participants at baseline (N=64).

Variable			Total (N=64)		Intervention group (n=32)		Control group (n=32)		*P* value	
Age (years), mean (SD; range)			55.6 (14.4; 20-82)		56.3 (15.6; 20-82)		54.9 (12.8; 20-82)		.44^a^	
**Gender, n (%)**										.20^b^
	Male	52 (81.3)		28 (87.5)		24 (75)				
	Female	12 (18.3)		4 (12.5)		8 (25)				
**Employment status, n (%)**										.79^b^
	Employed	41 (64.1)		20 (62.5)		21 (65.6)				
	Unemployed	23 (35.9)		12 (37.5)		11 (34.4)				
Monthly family income (BDT; US $1=BDT 123), mean (SD; range)			N/A^c^		33,250 (24,352.7; 5000-200,000)		33,750 (36,961.2; 5000-200,000)		.49ᵃ	
**Education status, n (%)**										>.99^d^
	Primary (5 years of schooling)	22 (34.4)		11 (34.4)		11 (34.4)				
	Secondary (10 years of schooling)	26 (40.6)		13 (40.6)		13 (40.6)				
	College and above (>10 years of schooling)	16 (25)		8 (25)		8 (25)				
**Religion, n (%)**										.30ᵇ
	Muslim	60 (93.8)		31 (96.9)		29 (90.6)				
	Hindu	4 (6.3)		1 (3.1)		3 (9.4)				
**Residence, n (%)**										.07ᵇ
	Urban	55 (85.9)		30 (93.8)		25 (78.1)				
	Rural	9 (14.1)		2 (6.3)		7 (21.9)				
**BMI, n (%)**										.62^d^
	Normal weight	25 (39.1)		13 (40.6)		12 (37.5)				
	Overweight	35 (54.7)		16 (50)		19 (59.4)				
	Obese	4 (6.3)		3 (9.4)		1 (3.1)				
**Modified Rankin Scale, n (%)**										.44^d^
	2	21 (32.8)		8 (25)		13 (40.6)				
	3	32 (50)		18 (56.3)		14 (43.8)				
	4	11 (17.2)		6 (18.8)		5 (15.6)				
**Smoking, n (%)**										0.30ᵇ
	Yes	24 (37.5)		10 (31.3)		14 (43.8)				
	No	40 (62.5)		22 (68.7)		18 (56.2)				
**Stroke type, n (%)**										.06ᵇ
	Ischemic	43 (67.2)		25 (78.1)		18 (56.3)				
	Hemorrhagic	21 (32.8)		7 (21.9)		14 (43.8)				
**Time of stroke, n (%)**										.18ᵇ
	First stroke	43 (67.2)		19 (59.4)		24 (75)				
	Recurrent stroke	21 (32.8)		13 (40.6)		8 (25)				
**Comorbidities, n (%)**										
	Hypertension	60 (93.8)		30 (93.8)		30 (93.8)		>.99ᵇ		
	Diabetes mellitus	50 (78.1)		25 (78.1)		25 (78.1)		>.99ᵇ		
	Arrhythmia	19 (29.7)		12 (37.5)		7 (21.9)		.17ᵇ		
	Chronic kidney disease	1 (1.6)		0 (0)		1 (3.1)		.31ᵇ		
	Obesity	4 (6.3)		3 (9.4)		1 (3.1)		.30ᵇ		
	Others^e^	10 (15.6)		4 (12.5)		6 (18.8)		.49ᵇ		

^a^Mann-Whitney *U* test.

^b^Chi-square test.

^c^N/A: not applicable.

^d^Fisher exact test.

^e^Others: such as meningitis, uterine tumor, and breast cancer.

### Outcomes

#### FIM

At baseline (T0), the mean FIM scores were 43.78 (SD 15.94) for IG and 43.79 (SD 14.10) for CG, with no significant difference (*P*>.99). FIM scores improved chronologically in both IG and CG, showing statistically significant improvements within groups; however, those changes were not statistically significant between groups or in the interaction (Table 2).

**Table 2:** Comparison of study outcomes between intervention and control groups at 4 time points.

		Baseline (T0), mean (SD)	After 1 month (T1), mean (SD)	After 2 months (T2), mean (SD)	After 3 months (T3), mean (SD)	*F* test (*df*)		*P* value	
**Functional Independence Measure^a^**							37.23 (1)^b^		<.001^b^
	Intervention group	43.8 (15.9)	64.4 (24.7)	73.5 (33.5)	78.4 (37.6)				
	Control group	43.8 (14.1)	64.3 (30.4)	67.7 (31.8)	74.3 (35.4)				
**Self-efficacy^c^**							0.046 (5)^d^		.83^d^
	Intervention group	23.9 (3.3)	N/A^e^	N/A	27.0 (6.9)				
	Control group	24.1 (4.6)	N/A	N/A	26.58 (7.14)				
**Social participation^c^**							0.272 (6)^d^		.61^d^
	Intervention group	39.1 (13.2)	N/A	N/A	30.4 (21.4)				
	Control group	36.2 (13.2)	N/A	N/A	28.2 (17.2)				
**Care burden^c^**							0.046 (5)^d^		.83^d^
	Intervention group	31.4 (4.6)	N/A	N/A	24.9 (13.6)				
	Control group	29.9 (6.4)	N/A	N/A	25.2 (12.6)				

^a^Functional Independence Measure was analyzed using a 2-way repeated-measures ANOVA. Interaction effect: *F*_3_=0.386; *P*=.60; between-group effect: *F*_1_=0.113; *P*=.74; within-group effect: *F*_1_=37.23; *P*<.001.

^b^2-way repeated measure ANOVA.

^c^Independent *t* test was used to compare groups.

^d^2-way ANOVA.

^e^N/A: not applicable.

#### Self-Efficacy

At baseline (T0), self-efficacy scores of both groups showed a mean of 23.95 (SD 3.33) for IG and 24.12 (SD 4.59) for CG (*P*=.89). After 3 months (T3), IG showed a higher mean (IG: mean 27.04, SD 6.95; CG: mean 26.58, SD 7.14) than CG; however, the difference was not significant (*P*=.82; Table 2).

#### Social Participation

At baseline (T0) and end line (T3), the mean restriction in social participation was 39.13 (SD 13.17) and 30.35 (SD 21.44), respectively, for the IG and 36.17 (SD 13.18) and 28.21 (SD 17.19), respectively, for the CG. However, the differences were not significant (*P*=.45 and *P*=.71) at both points (Table 2).

#### Caregiver Burden

The means of caregiver burden were 31.35 (SD 4.5) for IG and 29.88 (SD 6.39) for CG (*P*=.37) at baseline. At end line (T3), the means were 24.91 (SD 13.58) for IG and 25.21 (SD 12.60) for CG (*P*=.94), and no significant differences were found (Table 2).

#### FIM Subdomain Scores

FIM domain scores were analyzed at baseline (T0) and end line (T3) between the intervention and CGs. There were no statistically significant differences between the groups at both time points in the following categories: self-care, sphincter control, mobility, locomotion, communication, social cognition, and overall FIM (all *P*>.05; Multimedia Appendix 5).

Regarding FIM scores, the CG demonstrated improvements comparable to those of the IG. To explore the reasons, we conducted further analyses and found that participants in both groups engaged in private rehabilitation services in addition to our intervention. As this was not part of the exclusion criteria, and for ethical reasons, we could not restrict their access to such services. We therefore examined how these additional rehabilitation activities influenced our results. Table 3 presents the FIM score changes by service in each group. The changes were inconsistent and showed no clear relationship with the services received.

To examine which additional rehabilitation services affected the FIM score, we conducted a general linear model with age, mRS, and residence as covariates and found no statistically significant effects**.**

Compared with the CG, participants in the IG showed no significant difference in outcomes (*B*=2.48, SE=10.04, 95% CI –17.21 to 22.16; Wald *χ*^2^_1_=0.06; *P*=.81). Similarly, home-based rehabilitation did not significantly predict the outcome relative to the research intervention (*B*=–2.64, SE=10.25, 95% CI –22.72 to 17.44; Wald *χ*^2^_1_=0.07; *P*=.80). CRP-based rehabilitation also did not differ significantly from the research intervention condition (*B*=17.22, SE=16.80, 95% CI –15.70 to 50.15; Wald *χ*^2^_1_=1.05; *P*=.31; Table 4).

**Table 3:** Comparison of rehabilitation services from baseline to end line (n=47).

			Participants, n (%)		Baseline (T0)									After 3 months (T3)										Difference (T3 – T0)									
					mean (SD)		*t* test (*df*)			*P* value			mean (SD)			*t* test (*df*)			*P* value			mean (SD)				*t* test (*df*)			*P* value				
**FIM^a^** **score (n=47)**								–0.002 (45)			>.99						0.388 (45)			.70							0.423 (45)			.67			
	Intervention group	23 (100)		43.8 (15.9)								78.4 (37.6)									34.6 (34.1)												
	Control group	24 (100)		43.8 (14.1)								74.3 (35.4)									30.5 (33.2)												
**Study intervention + home-based rehabilitation (n=22)**								0.733 (45)			.24						0.174 (45)			.87							–0.189 (45)			.85			
	Intervention group	8 (34.8)		50.0 (18.8)								79.5 (37.9)									29.5 (32.7)												
	Control group	14 (58.3)		44.4 (14.6)								76.6 (35.6)									32.3 (34.4)												
**Study intervention + CRP^b^** **/facility-based (n=5)**								0.284 (45)			.80						0.197 (45)			.86							0.089 (45)			.94			
	Intervention group	4 (17.4)		39.0 (18.9)								83.0 (40.8)									44.0 (30.1)												
	Control group	1 (4.2)		33 (N/A^c^)								74 (N/A)									41.0 (N/A)												
**Study intervention only^d^** **(n=20)**								–0.508 (45)			.62						0.303 (45)			.77							0.517 (45)			.61			
	Intervention group	11 (47.8)		41.0 (12.5)								75.9 (39.9)									34.9 (38.5)												
	Control group	9 (37.5)		44.1 (14.5)								70.6 (38.9)									26.4 (34.6)												

^a^FIM: Functional Independence Measure.

^b^CRP: Centre for the Rehabilitation of the Paralyzed.

^c^N/A: not applicable.

^d^Numbers of “study intervention only” participants were those who did not receive either home-based rehabilitation or CRP/facility-based rehabilitation.

**Table 4:** Parameter estimates for group and rehabilitation type predicting the outcome variable.

Variable and category			*B* (SE)		95% CI		Wald chi-square (*df*)		*P* value
**Group (reference: control group)**									
	Intervention group	2.48 (10.04)		–17.21 to 22.16		0.061 (1)		.81	
**Rehabilitation type (reference: standard research intervention)**									
	Home-based rehabilitation	–2.64 (10.25)		–22.72 to 17.44		0.066 (1)		.80	
	CRP^a^ rehabilitation	17.22 (16.80)		–15.70 to 50.15		1.05 (1)		.31	

^a^CRP: Centre for the Rehabilitation of the Paralyzed.

#### Use of Assistive Devices

Participants in the IG were significantly used in assistive devices (*P*<.001), with 21 (91%) in the IG compared to only 4 (17%) in the CG. These findings indicate that the intervention was associated with significantly greater adoption of assistive devices. In CG, participants purchased assistive devices (exercise ball and TheraBand) with the advice of their physical therapist or outside rehabilitation services.

#### Extremity Impairments

All (n=47) participants had extremity impairments, and 94% (44/47) had both upper and lower extremity impairments, and 6% (n=3) had only upper extremity impairments. There was no significant difference observed between groups regarding extremity impairment (*P*>.99).

### Qualitative Findings

#### Overview

Among all the participants (n=32) who received the intervention, 23 were interviewed and observed for qualitative assessment. The qualitative data revealed 7 subthemes encompassing three key themes: (1) patients’ adherence to rehabilitation, (2) improvement of physical functioning and independence, and (3) perceived improvement by patients and family caregivers. Each theme demonstrated positive transformation between the first month and the third month, supported by participants’ perceptions and direct quotes (Multimedia Appendix 6).

#### Theme 1: Patients’ Adherence to Rehabilitation

##### Acceptance and Motivation to Use Assistive Devices

During the first month, participants were in the process of adapting to assistive devices and integrating them into their daily life activities. By the end of the third month, most had accepted and used these devices independently without any assistance from caregivers, despite emotional and financial challenges.

My family spends more on me than they earn

Male, 48 years, Case #2

These devices feel like blessings, but sometimes I lose hope

Female, 60 years, Case #6

##### Following the Exercise Regimen

Participants initially depended on caregivers to carry out suggested exercises; however, they consistently followed rehabilitation guidelines independently (at least 2 times per day for 30 minutes) in later times. This shift illustrates increased motivation and adherence to recommended regimens of rehabilitation.

#### Theme 2: Improvement of Physical Functioning and Independence

##### Mobility and Balance

At baseline, participants required assistance for walking and balance from caregivers. After intervention, most had the ability to ambulate with minimal assistance or modified independence using assistive devices by their own efforts.

Nowadays I’m feeling good. I’m going out with a walking cane and walking on my own. My family is relieved

Male, 20 years, Case #14

This quote demonstrates regained confidence and functional ability.

##### ADL

Dependence on daily tasks reduced progressively, from total or moderate dependence to moderate or minimal levels. Initially, they used assistive devices to perform their ADL. Gradually, after gaining the ability, they reduced their use of assistive devices.

I do everything by myself (with the help of assistive devices), whatever I can

Male, 65 years, Case #7

Now I can do my daily activities like eating and going to the toilet on my own. I just need a little help, but not always

Male, 48 years, Case #22

##### Comorbidities and Risk Factors

Participants commonly experienced comorbidities such as diabetes, hypertension, and visual disturbances. While persistent, most reported reduced symptom severity over time.

Participants received health education to combat comorbidities. One participant with diabetes and hypertension felt better after receiving our intervention. He explained:

I’m seeing blurry, specially at night... headaches appear when I go to sleep

Male, 20 years, Case #14

#### Theme 3: Perceived Improvement by Patients and Family Caregivers

##### Physical Improvement

Participants’ perceptions shifted from no or moderate improvement to substantial progress and functional independence using assistive devices. Another participant expressed:

I feel like I am improving day by day... this progress makes me feel very happy and hopeful

Male, 48 years, Case #2

##### Social Interaction and Participation

Initially, participants were socially withdrawn; they actively reengaged in community and family activities. After our intervention, they engaged more in social gatherings.

I go out and meet people. Being active has improved my condition

Male, 65 years, Case #21

This reflects enhanced self-efficacy and psychosocial well-being.

## Discussion

### Principal Findings

This study evaluated the feasibility of a nurse-led rehabilitation education with an assistive device to improve functional independence for self-care management, self-efficacy, social engagement, and reduction of caregiver burden among patients with poststroke disabilities. Over the 3-month study period, there was improvement after intervention; however, no significant differences were observed in any of the outcomes between the intervention and CGs.

### Sociodemographic Characteristics

The participants’ average age was 50 (range 20-82) years, which is consistent with the demographics of patients with stroke in LMIC, where strokes typically occur in early life compared to high-income countries [2]. Among the participants, male individuals were higher, which indicates that stroke is more common in men worldwide [37]. Therefore, it is essential to explore the underlying factors and comorbidities prevalent among male patients after stroke in that age group.

The majority of individuals came from families with moderate income or were employed, and educational attainment was consistent between groups. According to previous studies, socioeconomic and educational characteristics have an impact on stroke outcomes because decreased likelihood of receiving rehabilitation services and a worse long-term prognosis are associated with lower income and educational attainment [38].

The most prevalent comorbidities were diabetes mellitus and hypertension, which were found in almost a third of both groups, which are recognized risk factor profiles for stroke in Southeast Asian populations [39]. In the IG, we observed more ischemic strokes compared to the CG. As the prognosis of ischemic stroke is limited after intervention, this might affect our intervention, making it nonsignificant. Moreover, compared with ischemic strokes, hemorrhagic strokes are typically associated with higher early morbidity and mortality, resulting in more deaths, as reported in a previous study [40]. Despite persistent comorbidities, participants described adaptation and symptom control over time. This indicates resilience and coping strategies that help patients after stroke to survive with multimorbidity [7,41].

### Functional Independence

We did not find any difference in FIM scores over time between the groups. However, if we observe the trend, the FIM scores improved at each time point for both groups. In the IG, we assumed that the FIM scores were increased due to our intervention. We explored the cause of the improvement in FIM scores in the CG and found that they received rehabilitation services from external sources (home-based and CRP), which we could not inhibit due to ethical considerations. CRP provides rehabilitation services with a very highly trained professionals using all conventional therapies (eg, physiotherapy, occupational, speech, and language) in our study areas. Therefore, we can consider our intervention to be effective in 1 arm. This study emphasizes the importance of adequate rehabilitation intervention for patients with poststroke disabilities. Other studies also explained that poststroke disability can be improved after health education and the use of assistive devices [42]. Though our short (3 months) study duration shows FIM improvement in individual groups, one study showed no significant differences in FIM evolution when compared to usual care alone during the short period of time (<10 weeks) [43]. Improvements in mobility, balance, and ADL performance reflect global evidence that structured poststroke rehabilitation fosters early recovery within 3-6 months [44]. The progression from dependence to modified independence, as reported by participants, reinforces the importance of consistent physiotherapy and guided home exercises [9,45]. Our study findings highlight the significance of comprehensive standard care following a poststroke disability.

### Self-Efficacy, Social Participation, and Caregiver Burden

With respect to self-efficacy, social participation, and caregiver burden, we did not find any difference between the groups over time. To improve the self-efficacy of patients with stroke after discharge, we provided organized interaction and instruction that helped patients feel more confident and develop the skills they need to manage the aftermath of stroke. As in the CG, some of the patients received group interaction, education on health care and rehabilitation, and a community reintegration program from the CRP; therefore, we found improvement of self-efficacy in both groups. Recent research supports the importance of fostering self-efficacy through community services and standard care in patients, along with natural psychological adaptation [44].

The improvement of social participation and the decrease of caregiver burden were also observed in the IG and in the CG by the influence of CRP services. Despite prominent functional improvements, many patients after stroke report limitations in their ability to participate in the community, which raises concern [46]. Caregivers gradually adjust and become more confident as in the poststroke period, patients regain functioning ability, and families gain experience over time [47]. However, the persistently high levels of burden reported in recent surveys suggest that more focused and direct interventions, such as social support and behavioral education programs, may be effective [47].

The qualitative findings demonstrate how structured therapy promotes progressive recovery of patients after stroke, demonstrating significant gains in functional independence, adherence, and social reintegration. Additionally, patient narratives enhance comprehension by clarifying contextual and personal factors influencing rehabilitation involvement.

Over time, patients demonstrated more motivation and continued use of assistive devices, which is consistent with earlier studies that focused on gradual adaptation to rehabilitation services [44,48]. However, participants acknowledged that financial and caregiving burdens highlight socioeconomic challenges common in low-resource settings [17,42]. These results are consistent with research indicating that long-term rehabilitation success depends critically on patient motivation and family support [18].

This study shows that nurse-led rehabilitation enhances psychological well-being and quality of life through perceived physical improvement and increased social engagement [44]. Self-efficacy’s involvement in fostering recovery is supported by the expressed sense of hope and autonomy [21].

In resource-limited environments, barriers such as inadequate infrastructure, caregiver burden, and financial restraints reduce sustained engagement in rehabilitation [17,49]. These insights highlight the necessity of nurse-led, family-centered, community-based rehabilitation programs that tackle socioeconomic and clinical challenges [21].

Overall, the results show that, even in low-income settings, structured rehabilitation helps stroke survivors recover both physically and psychologically. To improve long-term adherence and social reintegration, future interventions should include systems of social support and monetary assistance.

### Strengths and Limitations

This is the first nurse-led randomized controlled trial in Bangladesh to improve patients’ ADL with disability by providing rehabilitation intervention. This study represents a proper allocation of participants through successful randomization. We provided assistive devices, education booklets, and follow-up through home visits. We carefully assessed patients and provided the appropriate assistive devices, ensuring that their usage was accurately measured. As this is a feasibility study, we observed that both the device and nurse education were well accepted by patients and family caregivers, indicating that this program could be feasibly implemented in the Bangladesh context.

Nevertheless, limitations must be acknowledged. High early mortality and attrition reflect the acute severity of illness in the study population. It causes underpowering of our study sample size. We conducted our study for a short period (3 months) of time with a small sample, which made it very difficult to interpret the findings. The study was conducted only in urban areas, which did not reflect the scenario of the whole country, which may limit generalizability. Further studies with multiple sites are needed to prevent participant contamination. More accurate measurements, rather than FIM, should be implemented to assess the influencing factors for self-care management. We also need to reconsider the mRS level (mRS-4) as there is a more severe disability that limits physical improvements.

### Conclusions

In conclusion, this is the first nurse-led pilot randomized controlled trial aimed at improving the functional abilities of patients with poststroke disabilities in Bangladesh. However, our rehabilitative intervention (3 months) showed no impact on FIM, self-efficacy, social participation, and caregivers’ burden for patients after stroke; obvious improvements were observed at each time point (baseline, monthly, and end line) of evaluation for both IG and CG. We assumed that this improvement occurred in the IG due to our intervention, and for the CG, improvement was observed due to the influence of rehabilitation received from home-based and institutional services. The result suggested that a comprehensive rehabilitation intervention is essential for the physical improvement of patients with poststroke disabilities. A future long-term interventional study with a large sample size will be guaranteed for better outcomes, considering all external influences to understand the underlying factors for the improvement of rehabilitative patients after stroke. Strengthening primary care facilities can be a strategic way for the community integration of patients with stroke disability. A government-led, systemic approach is essential to translate policy commitments into equitable access to rehabilitation services for all patients after stroke.

## Appendix

Multimedia Appendix 1 CONSORT eHEALTH checklist.

Multimedia Appendix 2 Contents of health booklet.

Multimedia Appendix 3 Qualitative interview guide.

Multimedia Appendix 4 Correlation of sociodemographic and preintervention Functional Independence Measure.

Multimedia Appendix 5 Functional Independence Measure subdomain analysis at baseline and endline between groups.

Multimedia Appendix 6 Qualitative content analysis.

## Abbreviations

ADL: activities of daily living
CG: control group
CONSORT: Consolidated Standards of Reporting Trials
CRP: Centre for the Rehabilitation of Paralyzed
FIM: Functional Independence Measure
ICF: International Classification of Functioning, Disability, and Health
IG: intervention group
LMIC: low- and middle-income countries
mRS: modified Rankin scale
NINS&H: National Institute of Neurosciences & Hospital
RA: research assistant
WHO: World Health Organization

## Data Availability

The data supporting the findings of this study are available on request from the corresponding authors. The data are not publicly available due to privacy and ethical restrictions.
