# Supplementary material for: Introduction of Nurse-Led Rehabilitation Services for Patients With Stroke After Discharge to Improve Self-Care Management in Bangladesh: Pilot Randomized Controlled Trial
Source: JMIR Rehabil Assist Technol. 2026 Jul 17;13:e88808. doi: 10.2196/88808 (PMC13428202; doi:10.2196/88808)
Supplement: Multimedia Appendix 2 [file rehab_v13i1e88808_app2.docx]

**Table: Contents of health booklet**

| **Serial** | **Contants** |
| --- | --- |
| 1 | Home environmental adjustment and modification (e.g. humidity, ventilation, remove unnecessary household equipment, arrange daily necessary utensils at bedside table) |
| 2 | Eating (eating position, food contents), practice and mouth care |
| 3 | Grooming and dressing guide |
| 4 | Bathing instructions |
| 5 | Toileting (bladder and bowel management) instructions |
| 6 | Movement or locomotion (turning over, sitting position, getting up in bed, methods of assistance by caregivers, standing up bedside, getting up from the floor, forward and backward movement) |
| 7 | Tips to avoid falling |
| 8 | Instructions for using assistive devices |
| 9 | Changing habits (Tips to avoid constipation, quit smoking, avoid alcohol, and food pyramid management in daily life) |
| 10 | Health monitoring (e.g. blood pressure, weight, blood sugar, medication intake) |
| 11 | Strategies to cope with stress |
| 12 | Exercise (both upper and lower extremities for improving muscle strength and preventing contracture) |
| 13 | Daily health record chart (Exercise and medication intake) |
