# Supplementary material for: Introduction of Nurse-Led Rehabilitation Services for Patients With Stroke After Discharge to Improve Self-Care Management in Bangladesh: Pilot Randomized Controlled Trial
Source: JMIR Rehabil Assist Technol. 2026 Jul 17;13:e88808. doi: 10.2196/88808 (PMC13428202; doi:10.2196/88808)
Supplement: Multimedia Appendix 3 [file rehab_v13i1e88808_app3.docx]

Table: Qualitative interview guide

| **Interview questions and point of observation** |
| --- |
| 1. Is patient using assistive devices at home? How are they adjusting with devices? |
| 1. Is the patient facing any difficulties using the assistive devices? |
| 1. How patient adjusting to daily life after having stroke? |
| 1. What are the risk factors after discharge at home? |
| 1. How much stroke impact the lifestyle of participants? |
| 1. What kind pf assistance the patient requires to maintain their daily life? |
| 1. How and what extent people adhere to the rehabilitation services and devices? |
| 1. How participants communicate with others? |
| 1. How they participate in social life after discharge at home? |
| 1. What is the mental status of the patient after having stroke? |
| Comments and remarks: |
