# Supplementary material for: Introduction of Nurse-Led Rehabilitation Services for Patients With Stroke After Discharge to Improve Self-Care Management in Bangladesh: Pilot Randomized Controlled Trial
Source: JMIR Rehabil Assist Technol. 2026 Jul 17;13:e88808. doi: 10.2196/88808 (PMC13428202; doi:10.2196/88808)
Supplement: Multimedia Appendix 4 [file rehab_v13i1e88808_app4.docx]

**Supplemental Table 1.** The correlations between sociodemographic and clinical characteristics with baseline

| **Variables** | **FIM score pre-intervention** | | |
| --- | --- | --- | --- |
|  | **M±SD** | **F/r/t** | **P value** |
| **Age** |  | -0.398 | .002 |
| **Gender** | | | |
| Male | 41.9±15.7 | -1.748 | 0.08 |
| Female | 49.1±12.9 |  |  |
| Monthly Family income |  | 0.224 | 0.75 |
| **Educational Status** | | | |
| Primary | 43.1±16.7 | 0.143 | 0.931 |
| Secondary | 43.1±14.0 |  |  |
| College and more | 43.9±16.5 |  |  |
| **Religious status** | | | |
| Muslim | 42.0±14.9 | -2.27 | .02 |
| Hindu | 61.1±11.8 |  |  |
| **Residence** | | | |
| Urban | 45.6±15.1 | -3.37 | < .001 |
| Rural | 29.0±7.6 |  |  |
| **BMI** |  | 0.022 | 0.87 |
| **BMI Category** | | | |
| Normal weight | 43.4±15.4 | 0.231 | 0.89 |
| Overweight | 43.8±16.0 |  |  |
| Obese | 37.5±15.4 |  |  |
| **Modified Rankin Scale (mRS)** | | | |
| 2 | 53.7±14.2 | 23.497 | < .001 |
| 3 | 41.8±13.5 |  |  |
| 4 | 27.8±4.7 |  |  |
| **Tobacco Smoking** | | | |
| Yes | 38.3±14.1 | -2.166 | .03 |
| No | 46.3±15.4 |  |  |
| **Stroke category** | | | |
| Ischemic | 41.8±16.5 | -1.524 | 0.13 |
| Haemorrhagic | 46.2±12.5 |  |  |
| **Comorbidities** | | | |
| Hypertension, (%) | 43.5±15.4 | -0.333 | 0.74 |
| Diabetes mellitus, (%) | 43.3±14.4 | -0.358 | 0.72 |
| Arrhythmia, (%) | 40.0±13.5 | -1.022 | 0.31 |
| CKD | 23.0±0.0 | -1.599 | 0.11 |
| Obesity | 37.5±15.4 | -0.472 | 0.637 |
| Others | 46.1**±**20.0 | -0.333 | 0.739 |
| **Time of stroke** | | | |
| First stroke | 42.7±15.3 | -0.458 | 0.65 |
| Repeated stroke | 44.6±15.6 |  |  |

r=Sperman correlation, t- t-test, F-ANOVA
