# Supplementary material for: Introduction of Nurse-Led Rehabilitation Services for Patients With Stroke After Discharge to Improve Self-Care Management in Bangladesh: Pilot Randomized Controlled Trial
Source: JMIR Rehabil Assist Technol. 2026 Jul 17;13:e88808. doi: 10.2196/88808 (PMC13428202; doi:10.2196/88808)
Supplement: Multimedia Appendix 5 [file rehab_v13i1e88808_app5.docx]

**Supplemental Table 2:** FIM sub-domain analysis at baseline and endline between groups

|  | **Baseline (T0)** | | | **Endline (T3)** | | |  |
| --- | --- | --- | --- | --- | --- | --- | --- |
|  | **mean ± SD** | **t** | **p** | **mean ± SD** | **t** | **p** |  |
|  |  |  |  |  |  |  |  |
| 1. **Eating** |  |  |  |  |  |  |  |
| Intervention Group | 3.1 ± 1.0 | -.368 | .714 | 4.6± 1.9 | .048 | .962 |  |
| Control Group | 3.2 ± 1.2 |  |  | 4.6 ± 1.7 |  |  |  |
| 1. **Grooming** |  |  |  |  |  |  |  |
| Intervention Group | 2.9 ± .9 | .132 | .895 | 4.6 ± 2.0 | .417 | .679 |  |
| Control Group | 2.8 ± 1.0 |  |  | 4.3 ± 1.8 |  |  |  |
| 1. **Bathing** |  |  |  |  |  |  |  |
| Intervention Group | 2.8 ± 1.2 | -.258 | .798 | 4.5 ± 2.0 | .546 | .588 |  |
| Control Group | 2.9 ± 1.2 |  |  | 4.2 ± 1.9 |  |  |  |
| 1. **Dressing (Upper Body)** |  |  |  |  |  |  |  |
| Intervention Group | 2.7 ± .8 | .467 | .642 | 4.5 ± 2.0 | 1.147 | .258 |  |
| Control Group | 2.6 ± .9 |  |  | 3.8 ± 2.0 |  |  |  |
| 1. **Dressing (Lower Body)** |  |  |  |  |  |  |  |
| Intervention Group | 2.4 ± .9 | .687 | .496 | 4.5 ± 2.1 | 1.503 | .140 |  |
| Control Group | 2.2 ± .9 |  |  | 3.5 ± 2.2 |  |  |  |
| 1. **Toileting** |  |  |  |  |  |  |  |
| Intervention Group | 2.3 ± 1.2 | .852 | .399 | 4.4 ± 2.2 | 1.171 | .248 |  |
| Control Group | 2.0 ± 1.0 |  |  | 3.6 ± 2.2 |  |  |  |
| **Total Self-Care** |  |  |  |  |  |  |  |
| Intervention Group | 16.2 ± 5.4 | .240 | .811 | 27.0 ± 12.1 | .857 | .396 |  |
| Control Group | 15.8 ± 5.5 |  |  | 24.0 ± 11.6 |  |  |  |
| 1. **Bladder management** |  |  |  |  |  |  |  |
| Intervention Group | 1.4 ± 1.1 | .066 | .948 | 4.1 ± 3.0 | -.146 | .885 |  |
| Control Group | 1.4 ± .8 |  |  | 4.2 ± 2.8 |  |  |  |
| 1. **Bowel Management** |  |  |  |  |  |  |  |
| Intervention Group | 1.39 ± .99 | .065 | .949 | 4.1 ± 3.0 | -.146 | .885 |  |
| Control Group | 1.38 ± .71 |  |  | 4.2 ± 2.8 |  |  |  |
| **Sphincter control Total** |  |  |  |  |  |  |  |
| Intervention Group | 2.8 ± 2.1 | .066 | .948 | 8.1 ± 5.9 | -.146 | .885 |  |
| Control Group | 2.8 ± 1.5 |  |  | 8.4 ± 5.5 |  |  |  |
| **Bed/ Chair/Wheelchair** |  |  |  |  |  |  |  |
| Intervention Group | 2.04 ± 1.19 | .126 | .900 | 4.1 ± 2.4 | .612 | .544 |  |
| Control Group | 2.0 ± 1.18 |  |  | 3.7 ± 2.3 |  |  |  |
| **Toilet** |  |  |  |  |  |  |  |
| Intervention Group | 2.0 ± 1.2 | .493 | .624 | 4.0 ± 2.3 | .548 | .586 |  |
| Control Group | 1.8 ± 1.1 |  |  | 3.6 ± 2.4 |  |  |  |
| **Bathtub/Shower** |  |  |  |  |  |  |  |
| Intervention Group | 2.0 ± 1.24 | .119 | .906 | 4.0 ± 2.3 | .458 | .586 |  |
| Control Group | 1.96 ± 1.16 |  |  | 3.6 ± 2.4 |  |  |  |
| **Mobility Total** |  |  |  |  |  |  |  |
| Intervention Group | 6.0 ± 3.5 | .247 | .806 | 12.1 ± 7.0 | .570 | .571 |  |
| Control Group | 5.8 ± 3.4 |  |  | 10.9 ± 7.0 |  |  |  |
| **Walk/ Wheelchair** |  |  |  |  |  |  |  |
| Intervention Group | 2.0 ± 1.1 | .262 | .795 | 3.9 ± 2.4 | .583 | .563 |  |
| Control Group | 2.0 ± 1.1 |  |  | 3.5 ± 2.4 |  |  |  |
| **Stairs** |  |  |  |  |  |  |  |
| Intervention Group | 1.7 ± 1.1 | .237 | .814 | 3.7 ± 2.4 | .339 | .736 |  |
| Control Group | 1.6 ± .9 |  |  | 3.5 ± 2.4 |  |  |  |
| **Locomotion Total** |  |  |  |  |  |  |  |
| Intervention Group | 3.7 ± 2.1 | .262 | .795 | 7.6 ± 4.8 | .464 | .645 |  |
| Control Group | 3.6 ± 2.0 |  |  | 6.9 ± 4.8 |  |  |  |
| **Motor Subtotal Score** |  |  |  |  |  |  |  |
| Intervention Group | 28.7 ± 11.0 | .272 | .787 | 54.8 ± 29.0 | .556 | .581 |  |
| Control Group | 27.9 ± 9.8 |  |  | 50.3 ± 26.8 |  |  |  |
| **Comprehension** |  |  |  |  |  |  |  |
| Intervention Group | 3.04 ± 1.15 | -.831 | .410 | 4.7 ± 2.2 | -.365 | .717 |  |
| Control Group | 3.33 ± 1.24 |  |  | 4.9 ± 2.0 |  |  |  |
| **Expression** |  |  |  |  |  |  |  |
| Intervention Group | 2.96 ± 1.15 | -1.08 | .285 | 4.9 ± 2.2 | -.221 | .826 |  |
| Control Group | 3.33 ± 1.24 |  |  | 4.9 ± 2.0 |  |  |  |
| **Communication Total** |  |  |  |  |  |  |  |
| Intervention Group | 6.0 ± 2.3 | -.959 | .342 | 9.5 ± 4.4 | -.294 | .385 |  |
| Control Group | 6.7 ± 2.5 |  |  | 9.8 ± 3.9 |  |  |  |
| **Social interaction** |  |  |  |  |  |  |  |
| Intervention Group | 3.3 ± 1.4 | -.764 | .449 | 4.9 ± 2.1 | -.424 | .673 |  |
| Control Group | 3.6 ± 1.5 |  |  | 5.1 ± 2.0 |  |  |  |
| **Problem solving** |  |  |  |  |  |  |  |
| Intervention Group | 2.9 ± 1.2 | -.623 | .537 | 4.6 ± 2.3 | -.344 | .733 |  |
| Control Group | 3.1 ± 1.5 |  |  | 4.8 ± 2.2 |  |  |  |
| **Memory** |  |  |  |  |  |  |  |
| Intervention Group | 3.0 ± 1.3 | -.394 | .696 | 4.6 ± 2.3 | -.397 | .693 |  |
| Control Group | 3.1 ± 1.6 |  |  | 4.9 ± 2.3 |  |  |  |
| **Social Cognition Total** |  |  |  |  |  |  |  |
| Intervention Group | 9.1 ± 4.0 | -.609 | .545 | 14.1 ± 6.7 | -.389 | .699 |  |
| Control Group | 9.8 ± 4.4 |  |  | 14.8 ± 6.4 |  |  |  |
| **Cognition subtotal** |  |  |  |  |  |  |  |
| Intervention Group | 15. 1 ± 5.9 | -.772 | .444 | 23.6 ± 10.5 | -.355 | .724 |  |
| Control Group | 16.5 ± 6.6 |  |  | 24.7 ± 10.2 | |  |  |
