# Supplementary material for: Introduction of Nurse-Led Rehabilitation Services for Patients With Stroke After Discharge to Improve Self-Care Management in Bangladesh: Pilot Randomized Controlled Trial
Source: JMIR Rehabil Assist Technol. 2026 Jul 17;13:e88808. doi: 10.2196/88808 (PMC13428202; doi:10.2196/88808)
Supplement: Multimedia Appendix 6 [file rehab_v13i1e88808_app6.docx]

**Supplemental Table 5.** Qualitative content analysis

| **Theme** | **Subtheme** | **First visit (T0)** | **Last visit (T3)** |
| --- | --- | --- | --- |
| Patient adherence to rehabilitation | Acceptance and motivation to use assistive devices | Adaptation and implementation of assistive devices on a regular basis.  *“My family spends more on me than they earn.” (Male, 48 years, Case # 2)*  *"I'm trying my best to improve my condition. These devices feel like blessings, but sometimes I lose hope." (Female, 60 years, Case # 6)* | Accepted the devices and use those independently. |
|  | Follow the exercise regimen | Adapted to guided rehabilitative exercise. | Consistent with rehabilitation guidelines. |
| Improvement of physical functioning and independence | Mobility and balance | Need assistance for walking and balance.  *“I now feel like a burden. I cannot walk, work and contribute to the family”. (Male, 48 years, Case # 2)*  *“I'm worried about my disabled side. It seems it's taking too much time to improve.” (Male, 65 years, Case # 7)* | Gained the ability to walk with minimal assistance to modified independence.  *“Nowadays I'm feeling good. I'm going out and walking on my own. My family is relieved.” (Male, 20 years, Case # 14)* |
|  | Performing the Activity of Daily Living (ADL) | Total to moderate dependent for ADL | Moderate to minimal dependent for ADL.  *“I do everything by myself, whatever I can. I can move my left hand but cannot hold anything using my fingers. I feel weak on the left side”. (Male, 65 years, Case # 7)*  *“I am quite improved compared to the previous days. Now I can do my daily activities like eating and going to the toilet on my own. I just need a little help, but not always.” (Male, 48 years, Case # 22)* |
|  | Comorbidities and risk factors | Diabetes, breast cancer, left ventricular thrombus, bacterial meningitis, visual disturbance, hypertension, etc.  *“I'm seeing blurry specially at night. Also, the headaches appear when I go to sleep.” (Case # 14)*  *“I've been constipated for days. It's so hard for me to defecate.” (Case # 21)*  *“It feels like it's burning inside my chest. Sometimes I cannot breathe.” (Case # 22)* | Consistent with subsided |
| Perceived improvement by patients and family caregivers | Physical improvement | No improvement to moderate improvement.  *"I feel like I am improving day by day. I truly hope to recover completely soon, and this progress makes me feel very happy and hopeful."(Case # 2)* | Moderate to Total independence. |
|  | Social interaction/ participation | Less socially active. | Active involvement in social gatherings.  *"I go out and meet people. Being active has improved my condition”. (Case # 21)*  *"I go out in the afternoon near my house and meet my neighbours and my friends, which makes me feel good for the rest of the day.” (Case # 22)* |
